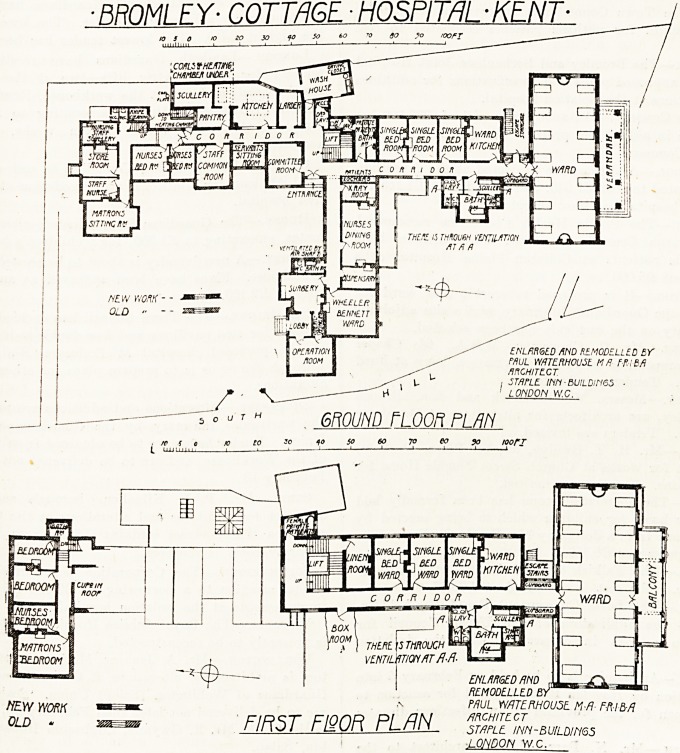# The Alterations to Bromley Cottage Hospital

**Published:** 1913-02-08

**Authors:** 


### HOSPITAL ARCHITECTURE AND CONSTRUCTION.

### The Alterations to Bromley Cottage Hospital.

THIS hospital was erected in 1875 and enlarged in 1896, 1900, and 1910. The original building was designed by Mr. E. Hellicar, and the last alterations in 1910-11 were carried out under Mr. Paul Waterhouse, M.A., F.R.I.B.A.

The plans show that the hospital has developed to an extent for which the word "cottage" is no longer appropriate. It is, in fact, to all intents and purposes a small general hospital, although the total number of beds, thirty-six (if we assume that the Wheeler Bennett Ward holds two beds), is below the usual limit of fifty.

The original building had no ward of greater capacity than five beds; and we think that Mr. Waterhouse exercised a sound judgment in advising that additional ward accommodation should take the form of two large wards rather than a number of small ones. The new wing is built on at the south end of the building, and contains a ward of fourteen beds on the ground floor, with a similar ward on the first floor. Each ward has a wide verandah on the south side, on to which patients can be wheeled in their beds.

The sanitary offices, which have to serve also for the existing three single-bed wards on each floor, have been built out on the west side of the corridor, and are ingeniously contrived in a somewhat restricted space. It may be objected that the orthodox cross-ventilated, or "cut-off," lobby is absent, and that a precaution for preventing the air from the sanitary offices passing into the building generally regarded as essential has been neglected. In reply we may point out that in the days when the "cut-off" lobby was invented the art of sanitary plumbing was practically non-existent; and the precaution was really necessary; and, as if to demonstrate the futility of the arrangement, it will almost invariably be found that the lobby doors are fixed open, if not permanently, at any rate when the offices are being used.

The chief alteration in addition to those referred to is a rearrangement of the kitchen offices, and the erection of a small mortuary in a corner of the site sheltered from the main buildings.

The building as it stands is an interesting example of the growth of a small cottage hospital into what may be regarded as an intermediate stage between the cottage and the general hospital type.

## Figures and Tables

**Figure f1:**